# MrMYB6 From Chinese Bayberry (*Myrica rubra*) Negatively Regulates Anthocyanin and Proanthocyanidin Accumulation

**DOI:** 10.3389/fpls.2021.685654

**Published:** 2021-06-18

**Authors:** Liyu Shi, Xin Chen, Kang Wang, Minjie Yang, Wei Chen, Zhenfeng Yang, Shifeng Cao

**Affiliations:** College of Biological and Environmental Sciences, Zhejiang Wanli University, Ningbo, China

**Keywords:** Chinese bayberry, anthocyanins, proanthocyanidins, repressor, MYB transcription factor

## Abstract

Anthocyanins and proanthocyanidins (PAs) are important flavonoids in Chinese bayberry (*Morella rubra*), which functions in fruit color and exhibits multiple health promoting and disease-preventing effects. To investigate the regulation of their biosynthesis in Chinese bayberries, we isolated and identified a subgroup 4 MYB transcription factor (TF), MrMYB6, and found MrMYB6 shared similar repressor domains with other MYB co-repressors of anthocyanin and PA biosynthesis after sequence analysis. Gene expression results revealed the transcripts of *MrMYB6* were negatively correlated with the anthocyanin and insoluble PA contents and also with the gene expressions involved in anthocyanin biosynthesis and PA specific genes such as *MrLAR* and *MrANR* during the late ripening stages of bayberries. In addition, overexpression of *MrMYB6* in tobacco inhibited the transcript levels of *NtCHI, NtLAR*, and *NtANR2*, resulting into a decline in the levels of anthocyanins and PAs in tobacco flowers. We further found that MrMYB6 interacted with MrbHLH1 and MrWD40-1 to form functional complexes that acted to directly repress the promoter activities of the PA-specific gene *MrLAR* and *MrANR* and the anthocyanin-specific gene *MrANS* and *MrUFGT*. Taken together, our results suggested that MrMYB6 might negatively regulate anthocyanin and PA accumulation in Chinese bayberry.

## Introduction

Flavonoids, widespread secondary metabolites, play many important roles in the development of plants (Winkel-Shirley, 2001). Anthocyanins and proanthocyanidins (PAs) are two major classes of flavonoids in fruits. The former is associated with the wide range of colors including bright orange, pink, red, violet, and blue in fruits, and the latter contribute to the astringency and flavor of fruits. Furthermore, both anthocyanins and PAs are considered as dietary antioxidants that are beneficial to human health in reducing free radical mediated injury and cardiovascular disease (Middleton et al., 2000). Therefore, they play important roles in influencing fruit quality.

The biosynthesis of anthocyanins and PAs share many enzymes in the upstream pathway, including chalcone synthase (CHS), chalcone isomerase (CHI), flavanone 3-hydroxylase (F3H), flavonoid 3′-hydroxylase (F3′H), and dihydroflavonol 4-reductase (DFR). Leucoanthocyanidins produced by DFR are the first branch point between the anthocyanin and PA biosynthesis pathways. Then leucoanthocyanidin reductase (LAR) and anthocyanidin synthase (ANS) can use leucoanthocyanidins to produce trans-flavan-3-ols and anthocyanidins, respectively. After that, anthocyanidins will be converted either to cis-flavan-3-ols by anthocyanidin reductase (ANR) or to anthocyanins by UDP-glucose flavonoid 3-O-glucosyltransferase (UFGT).

Increasing evidences have shown that the synthesis of anthocyanins and PAs is transcriptionally regulated by a conserved MBW complex that consists of R2R3-MYB, bHLH, and WD40 transcription factors (TFs) (Ramsay and Glover, 2005). Of these TFs, R2R3-MYB protein displays the functional specificity of the complex and determines which pathways are regulated. To date, many R2R3-MYB activators responsible for anthocyanin and PA accumulation have been characterized in various plants. For instance, PpMYB10.1 was able to activate anthocyanin biosynthesis and PpMYB7 regulated PA biosynthesis in peach (Rahim et al., 2014; Zhou et al., 2015). In apple, MdMYB1, MdMYB10, and MdMYB110a were activators responsible for anthocyanin accumulation (Takos et al., 2006; Espley et al., 2007; Chagne et al., 2013), while MdMYB9 and MdMYB11 played a positive role in regulating PA biosynthesis (Gesell et al., 2014; An et al., 2015). The accumulation of anthocyanins in grapevine was enhanced by VvMYBA1 and VvMYBA2 (Walker et al., 2007), whereas VvMYBPA1, VvMYBPA2, and VvMYBPAR promoted PA biosynthesis (Bogs et al., 2007; Terrier et al., 2009; Koyama et al., 2014).

Besides MYB activators, R2R3-MYB inhibitors are also important contributors to flavonoid regulation. Many MYB repressors, such as strawberry FaMYB1 (Aharoni et al., 2001), petunia PhMYB27 (Albert et al., 2014), peach PpMYB17-20 (Zhou et al., 2016), grapevine VvMYB4-like (Ricardo Perez-Diaz et al., 2016), apple MdMYB16 (Xu et al., 2017), and MdMYB15L (Xu et al., 2018), have been shown to play key roles in inhibiting anthocyanin biosynthesis. Grapevine VvMYBC2-L1 was identified as the first PA pathway repressor (Huang et al., 2014). Subsequently, some other R2R3-MYB repressors have been identified. For example, FtMYB8 from Tartary buckwheat inhibited both anthocyanin and PA biosynthesis, and interacted with AtTT8/FtTT8/FtGL3 in yeast (Huang et al., 2019). Overexpression of poplar *MYB182* reduced anthocyanin and PA accumulation, which depended on the interaction between MYB182 and bHLH131 (Yoshida et al., 2015). In *Medicago truncatula*, MYB2 was identified as an inhibitor of both anthocyanin and PA biosynthesis, binding to bHLH factors TT8 (Jun et al., 2015). Interestingly, all these repressors typically belonged to the subgroup 4 R2R3-MYB TFs (Kranz et al., 1998), and shared the C1 motif (LlsrGIDPxT/SHRxI/L) and C2 motif (pdLNLD/ELxiG/S) with a core consensus sequence of either LxLxL or DLNxxP in the C-terminal region, which were identified as repression domains (Dubos et al., 2010; Zhou et al., 2019).

Chinese bayberry (*Morella rubra* Sieb. et Zucc.) contains large amounts of anthocyanins and PAs, and is an important source of natural antioxidants (Bao et al., 2005; Shi et al., 2018a). To date, only one MBW transcription complex, MrMYB1-MrbHLH1-MrWD40-1, has been observed to promote fruit anthocyanin formation (Liu, X. et al., 2013; Liu, X. F. et al., 2013), however, there is no available information on TFs negatively regulating anthocyanin biosynthesis. Furthermore, the MYB TFs that specifically regulate PA biosynthesis in Chinese bayberry have not been reported yet. From the transcriptome data, our group previously isolated three putative subgroup 4 MYB repressors (*c24596_g1, c28754_g2*, and *c48297_g1*) from Chinese bayberry (Shi et al., 2018b). Here, we cloned and characterized *MrMYB6 (c24596_g1)* as the ortholog of other MYB TFs that have previously revealed as a negative controller of flavonoid biosynthesis in fruit species (Albert et al., 2014; Cavallini et al., 2015; Yoshida et al., 2015). Functional analysis indicated that MrMYB6 could regulate the transcripts of flavonoid biosynthesis genes, and its overexpression in tobacco repressed both anthocyanin and PA accumulation, suggesting that MrMYB6 might be an inhibitor of anthocyanin and PA synthesis in Chinese bayberry.

## Materials and Methods

### Plant Materials

Chinese bayberry (*Myrica rubra* Sieb. and Zucc. cv. Biqi) trees were planted in an orchard in Cixi, Zhejiang Province. Fruits were picked at 57, 71, 85, 99, and 113 days after full bloom (DAFB) from four trees. The mixed fruits from the four trees were divided into three biological replicates for each developmental stage. All samples were frozen rapidly in liquid nitrogen and kept at −80°C for further use. Tobacco plants (*Nicotiana tabacum* cv. Samsunand *Nicotiana benthamiana*) were planted in a greenhouse with a 16 h light/8 h dark photoperiod at 25°C.

### Anthocyanin and PA Analysis

Total anthocyanin content was determined using the method reported by our group (Shi et al., 2014). Total soluble PA content was measured by reaction with dimethylaminocinnamaldehyde (DMACA), and total insoluble PA content was evaluated according to the butanol-HCl method reported in our previous study (Shi et al., 2018a).

### Quantitative Real-Time PCR

Total RNA was obtained using a Plant RNA Kit (Omega, Norcross, GA) followed by treatment with RNase-free DNase I. First-strand cDNA synthesis was performed using the SuperRT First Strand cDNA Synthesis Kit (CWBIO, Beijing, China) following to the manufacturer's recommendations. qRT-PCR analysis was conducted using LightCycler 480 SYBR Green Master (Roche, Shanghai, China) on a Bio-Rad CFX96 Real-Time PCR System (BioRad, Hercules, CA, USA). Each reaction was conducted with a 12.5 μl reaction volume containing 6.25 μl of SYBR Green PCR Master Mix, 4.75 μl of RNase-free water, 0.5 μl of cDNA, and 0.5 μl of each primer (10 mM). The transcript levels were calculated using the 2^−Δ*ΔCt*^ method and normalized using the housekeeping gene *MrACT* (GQ340770) for Chinese bayberry and *NtACTIN* (GQ339768) for tobacco. All analyses were determined using three biological replicates with 40 fruits per replicate. Primers used for qRT-PCR are shown in Supplementary Table 1.

### Isolation and Sequence Analysis of MrMYB6

The 3′-end sequence of *MrMYB6* gene was amplified using the SMART RACE cDNA amplification kit (Clontech, Mountain View, CA, USA). Nested primers were designed based on the partial *MrMYB6* sequence found in the transcriptome data, and are shown in Supplementary Table 1 (Shi et al., 2018b). The product of PCR was recombined into pMD18-T cloning vector (TaKaRa, China). The deduced amino acid sequences of MrMYB6 and other R2R3-MYB repressors were aligned using the ClustalX2 program. Phylogenetic analysis was performed by the MEGA 7 program with the neighbor-joining (NJ) method and 1,000 bootstrap replicates.

### Subcellular Localization

The full-length *MrMYB6* coding region without the stop codon was amplified and ligated into the pCAMBIA1301-GFP vector using primers listed in Supplementary Table 1. After sequencing, the recombinant plasmid and the GFP control vector were electroporated into *Agrobacterium tumefaciens* strain EHA105, and the cultures were adjusted to an OD 600 of 0.5 with infiltration buffer (10 mM MES, 10 mM MgCl_2_, 150 mM acetosyringone, pH 5.6) and then infiltrated into tobacco (*N. benthamiana*) leaves using needleless syringes. The GFP fluorescence of the tobacco leaves was detected 3 days after infiltration using the A1+ confocal laser scanning microscope (Nikon, Tokyo, Japan).

### Transformation of Tobacco

The full-length *MrMYB6* coding region was inserted into the pCAMBIA-1300 binary vector using primers shown in Supplementary Table 1. The resulting binary vector was then transformed into *A. tumefaciens* strain EHA105 by electroporation. Transgenic tobacco plants overexpressing of *MrMYB6* were obtained using the leaf disc infection technology reported by our group (Horsch et al., 1985). The putative transformed plants were selected on MS medium containing 250 mg/L carbenicillin and 25 mg/L hygromycin and finally identified by qRT-PCR analysis.

### Yeast Two-Hybrid Assay

The possible interactions between proteins were tested in yeast using the Matchmaker® Gold Yeast Two-Hybrid System (Clontech, Japan). Full-length coding regions of Arabidopsis *AtTT8, AtTTG*, and bayberry *MrHLH1* and *MrWD40-1* were cloned into pGBKT7 (bait) vector, and the full-length *MrMYB6* was ligated into pGADT7 (prey) vector (primers are listed in Supplementary Table 1). Then, each pair of recombinant plasmids was co-transferred into Y2H yeast by the PEG/LiAC method. Yeast transformants were selected on SD/-Leu/-Trp medium and the interactions were detected on SD/-Leu/-Trp/His/-Ade medium in the presence of X-α-Gal. pGADT7-T co-transformed with pGBKT7-p53 or pGBKT7-Lam were used as positive or negative controls.

### Luciferase Activity Assay

The promoter regions of *MrANR* and *MrLAR* were isolated using the GenomeWalker^TM^ Universal Kit (Clontech, USA) according to the manufacturer's protocol, and the promoters of *MrANS* and *MrUFGT* were directly amplified based on the sequences reported (Liu, X. F. et al., 2013). These promoter regions were then ligated into the pGreenII 0800-LUC reporter vector. The full-length coding sequences of bayberry *MrMYB6, MrbHLH1, MrWD40-1*, and *Arabidopsis AtTT8, AtTTG* were amplified and cloned into the pGreenII 62-SK effector vector. Primer information is shown in Supplementary Table 1. These recombinant vectors were then electroporated into Agrobacterium strain EHA105. After cultivation, cells were resuspended in infiltration buffer (10 mM MgCl_2_, 10 mM MES, 150 mM acetosyringone, pH 5.6), adjusted to an OD 600 of 0.2, and incubated for 2 h at room temperature without shaking before infiltration. Four-week-old leaves of *N. benthamiana* were infected with mixed agrobacteria and collected for dual luciferase test according to the method described previously (Hellens et al., 2005).

## Results

### Cloning and Identification of *MrMYB6*

In our previous study, three putative subgroup 4 MYB repressors (*c24596_g1, c28754_g2*, and *c48297_g1*) of Chinese bayberry were obtained from the transcriptome data (Shi et al., 2018b). Here, we cloned and identified *MrMYB6 (c24596_g1)*, which was named based upon sequence homology to the genes found through the BLAST in the NCBI database, and the characterization of the other two MYB repressors will be carried out in future work. The 3′-end of *MrMYB6* open reading frame (ORF) was amplified via 3′RACE-PCR and an 879 bp fragment was obtained. Sequence analysis indicated *MrMYB6* contained an ORF of 684 bp encoding 228 amino acid resides with a predicted molecular mass of 25.72 kD and a calculated PI of 8.38.

The sequence alignment of MrMYB6 with MYB repressors of other species suggested that their N-terminus contained highly conserved R2R3 domain with a bHLH-binding domain (Grotewold et al., 2000) and their C-terminus included two conserved motifs of MYB subgroup 4 TFs, the C1 and C2 motif, of which the C2 motif associated with EAR repressor domain (Jin et al., 2000; Kagale and Rozwadowski, 2011; Shen et al., 2012) (Supplementary Figure 1A). MrMYB6 shared the LxLxL-type EAR motif with other subgroup 4 MYBs that inhibit both PA and anthocyanin biosynthesis, such as PtrMYB182 (Yoshida et al., 2015), VvMYBC2-L3 (Cavallini et al., 2015), FtMYB8 (Huang et al., 2019), and FaMYB1 (Aharoni et al., 2001; Paolocci et al., 2011). In addition, MrMYB6 contained a TLLLFR repressor motif, which has been identified in FaMYB1-like proteins such as PtrMYB182 (Yoshida et al., 2015) and VvMYBC2 (Cavallini et al., 2015), but not in AtMYB4-like repressors (Jin et al., 2000). Phylogenetic analysis showed that R2R3 MYB inhibitors were separated into two clades, AtMYB4-like and FaMYB1-like (Supplementary Figure 1B). MrMYB6 was clustered in FaMYB1-like clade, which contained co-repressors in anthocyanin and PA biosynthesis including VvMYBC2 from grapevine (Cavallini et al., 2015), PtrMYB182 from Poplar (Yoshida et al., 2015), PhMYB27 from petunia (Albert et al., 2014), FtMYB8 from buckwheat (Huang et al., 2019), MtMYB2 from Medicago (Jun et al., 2015), and FaMYB1 (Aharoni et al., 2001; Paolocci et al., 2011) from strawberry. Thus, MrMYB6 might act as a negative controller of flavonoid biosynthesis in Chinese bayberry.

### Expression Profiles and Subcellular Location of MrMYB6

**Figure 1:**
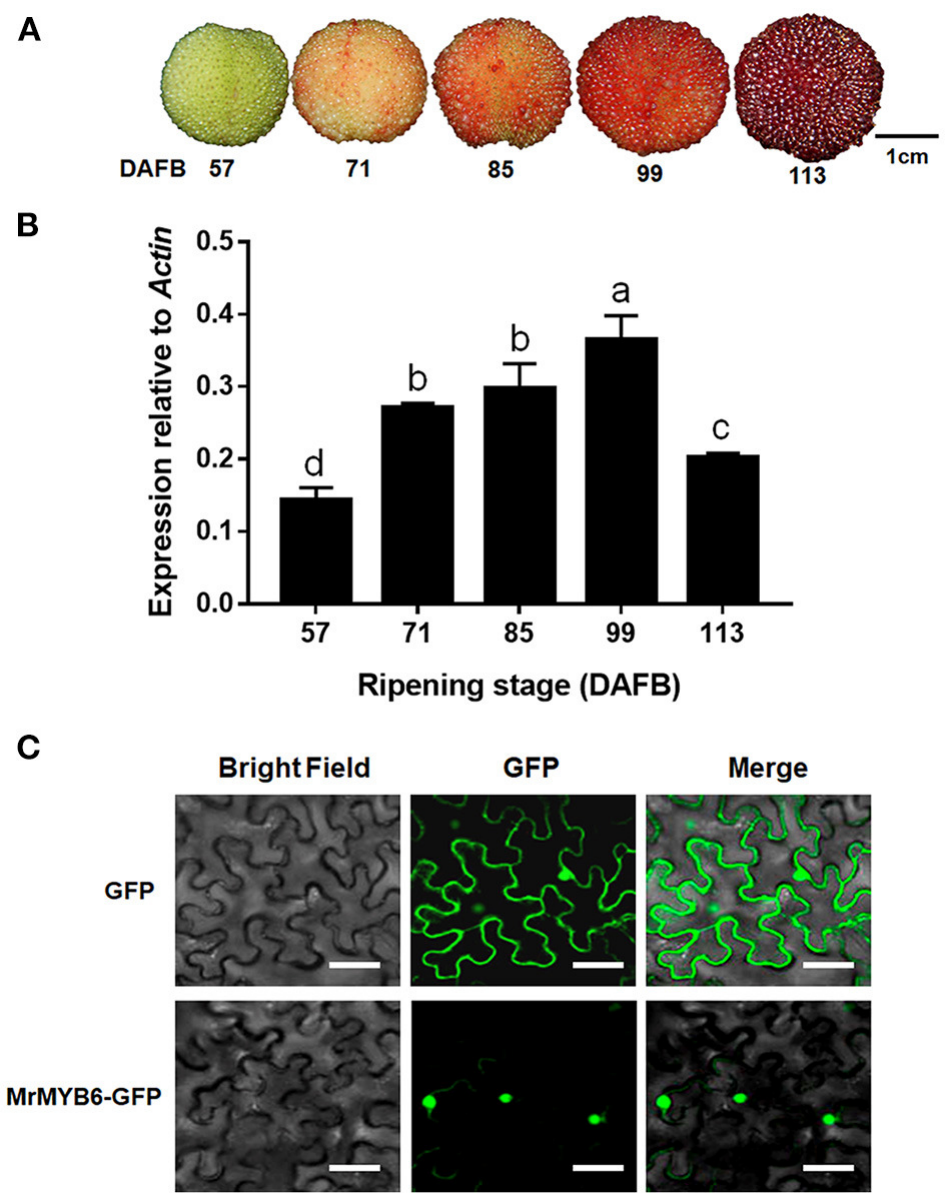
*MrMYB6* expression pattern during bayberry fruit development. **(A)** The bayberry fruits at 57, 71, 85, 99, and 113 days after full bloom (DAFB) used in this study. Scale bar represents 1 cm. **(B)** Expression levels of *MrMYB6* at different stages. The data were normalized to the *MrACT* expression level. Error bars indicate the mean ± *SE* of three replicate reactions. Letters (a, b, c, and d) represent significant difference between wild-type and transgenic plants according to the Duncan's multiple range test (*P* < 0.05). **(C)** Subcellular localization of *MrMYB6* in tobacco leaf epidermal cells. Scale bar represents 50 μm.

During fruit development, the red color of bayberry fruit gradually deepened and reached the deepest point at 113 day after full bloom (DAFB) (Figure 1A). *MYB6* gene exhibited low expression level at 57 DAFB and 113 DAFB, but was highly expressed from 71 DAFB to 99 DAFB (Figure 1B).

In order to investigate the subcellular location of MrMYB6, the 35S:MrMYB6-GFP fusion protein and the 35S:GFP control protein were transiently expressed in tobacco leaves. By scanning the GFP signal, it was found that the 35S:MrMYB6-GFP fusion protein was exclusively localized in the nucleus, whereas the control 35S:GFP was distributed in both the cytoplasm and the nucleus (Figure 1C).

### Overexpression of *MrMYB6* in Tobacco Represses Anthocyanin and PA Accumulation

**Figure 2:**
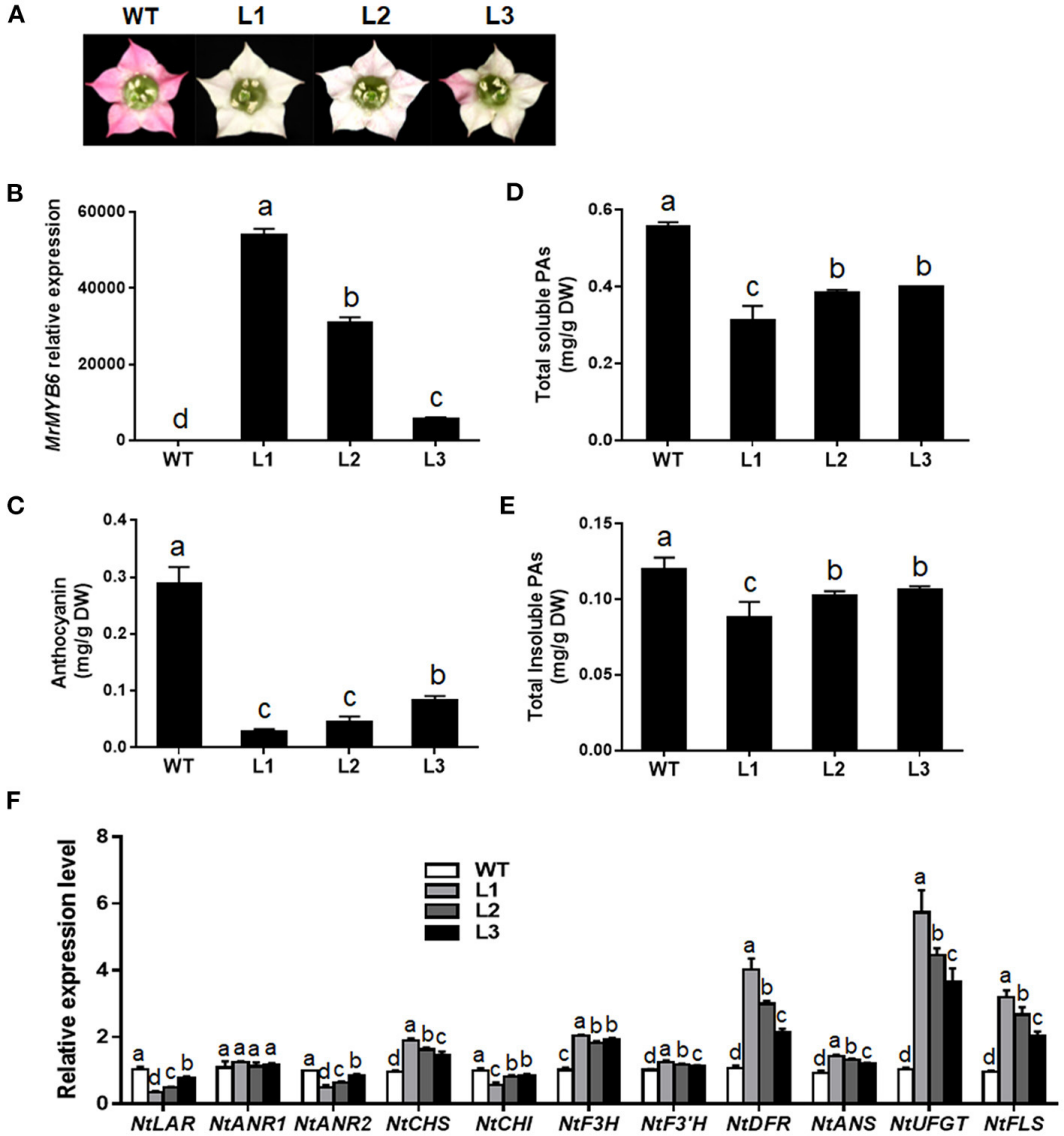
Ectopic expression of *MrMYB6* gene in tobacco. **(A)** Comparison of flower color in wild type (WT) and three *MrMYB6* transgenic (L1, L2, and L3) tobacco lines. **(B)** Relative expression levels of *MrMYB6* in transgenic flowers. The data were normalized using the WT sample and the *NtACTIN* (GQ339768) gene as the internal controls and calculated using the 2^−Δ*ΔCt*^ method. **(C)** Anthocyanin content in flowers of WT and three *MrMYB6* tobacco lines. **(D)** Total soluble PAs levels of WT and transgenic flowers. **(E)** Total insoluble PAs levels of WT and transgenic flowers. **(F)** Expression analysis of flavonoid biosynthetic genes in flowers of *MrMYB6* transgenic tobacco. Error bars indicate the mean ± *SE* of three replicate reactions. Letters (a, b, c, and d) represent significant difference between wild-type and transgenic plants according to the Duncan's multiple range test (*P* < 0.05).

To characterize the function of this TF, transgenic tobacco plants overexpressing *MrMYB6* were generated. From twelve independent T1 transgenic lines, nine plants showed weaker flower color than wild-type (WT) (date not shown). Among them, three transgenic lines (L1, L2, and L3) with obvious white colored flowers were selected for further study after confirmation of high *MrMYB6* transcript levels using qRT-PCR analysis (Figures 2A,B). The result of anthocyanin analysis showed that the anthocyanin content in flowers of all transgenic lines was significantly reduced compared with WT (Figure 2C). The soluble and insoluble PA levels in transgenic tobacco petals were also significantly declined (Figures 2D,E). In addition, all three *MrMYB6* overexpression lines exhibited decreased *NtLAR, NtANR2*, and *NtCHI* expression and increased *NtCHS, NtF3H, NtF3*′*H, NtDFR, NtANS, NtUFGT*, and *NtFLS* expression in transgenic flowers (Figure 2F).

### MrMYB6 Interacts With bHLH and WD40 TFs

Previous studies demonstrated that *Arabidopsis* AtTT2 formed the MBW complex with AtTT8 and AtTTG1 to promote PA accumulation (Baudry et al., 2004), and MrMYB1-MrbHLH1-MrWD40-1 complex was essential for anthocyanin biosynthesis in Chinese bayberry (Liu, X. F. et al., 2013; Liu, X. et al., 2013). Therefore, we carried out yeast two-hybrid assays to determine whether MrMYB6 interacts with AtTT8, AtTTG1, MrbHLH1, and MrWD40-1 to regulate the biosynthesis of PAs and anthocyanins.

**Figure 3:**
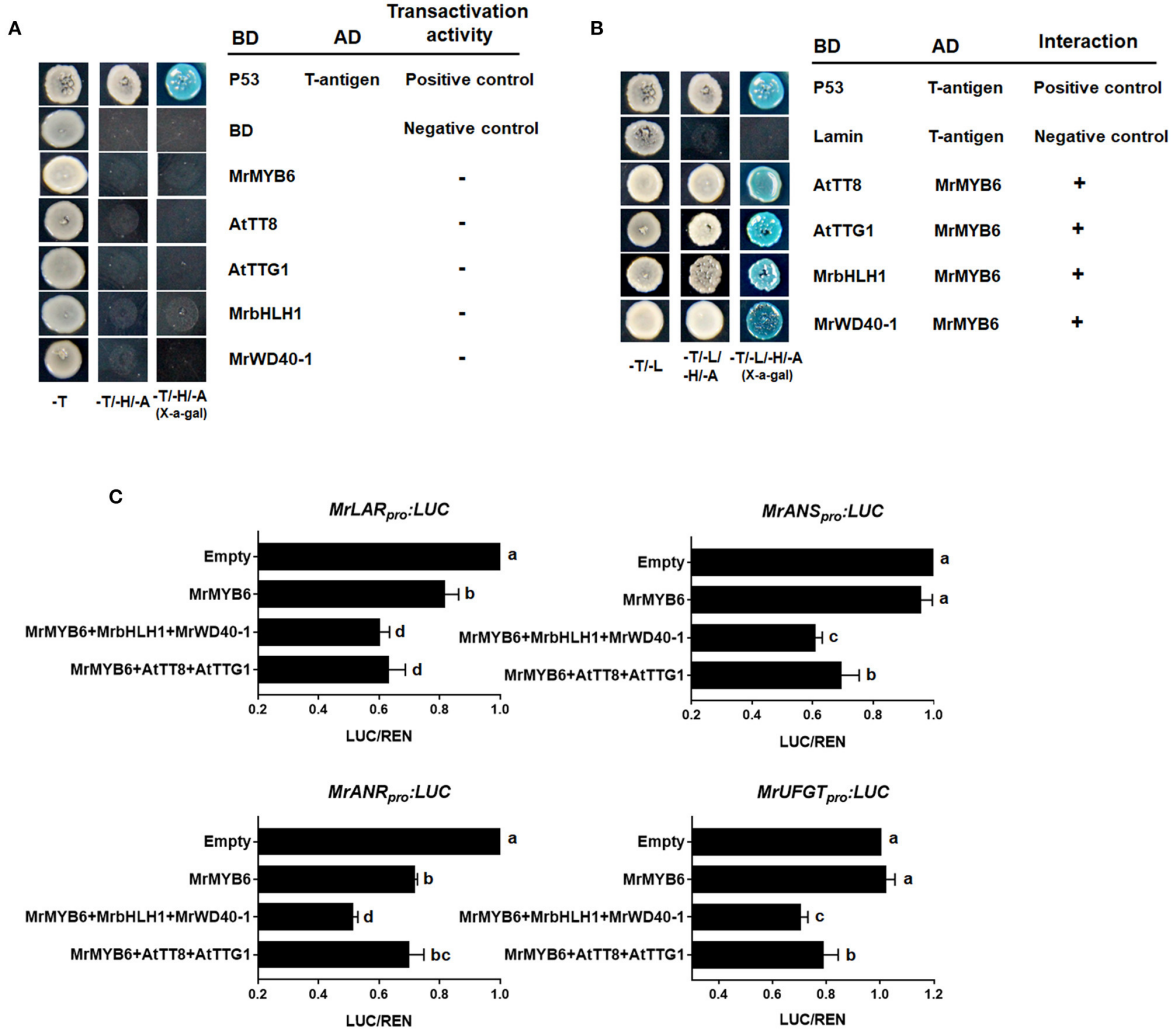
The role of MrMYB1-MrbHLH1-MrWD40-1 complex. **(A)** Autoactivation tests in yeast cells. **(B)** MrMYB6 interacts with AtTT8/AtTTG1/MrbHLH1/MrWD40-1 in yeast. -T, SD/-Trp medium; -T/-H/-A, SD/-Trp-His-Ade medium; -T/-L, SD/-Trp-Leu medium; -T/-L/-H/-A, SD/-Trp-Leu-His-Ade medium; BD, DNA-binding domain; AD, activation domain. **(C)** Transcriptional repression ability of MrMYB6 to the promoters of PA-specific gene *MrLAR* and *MrANR* and anthocyanin-specific gene *MrANS* and *MrUFGT*. The LUC/REN ratio of the empty vector plus promoter was set as 1. Different letters above the columns indicate significant differences at *p* < 0.05. Error bars indicate *SE* from four biological replicates.

As shown in Figure 3A, yeast transformants harboring the pGBKT7-MrMYB6/AtTT8/AtTTG1/MrbHLH1/MrWD40-1 constructs could not grow on SD/-Trp-His-Ade selection plates, indicating that these proteins had no self-activating activities. Nevertheless, the yeast cells possessing both pGADT7-MrMYB6 and pGBKT7-AtTT8/AtTTG1/MrbHLH1/MrWD40-1 grew well and showed α-galactosidase activity on the SD/-Trp/-Leu/-His/-Ade medium (Figure 3B). These results showed that MrMYB6 interacted physically with AtTT8, AtTTG1, MrbHLH1, and MrWD40-1 and implied that MrMYB6 might form a MBW complex to control PA and anthocyanin biosynthesis.

### MrMYB6 Inhibits the Promoters of Anthocyanin and PA Pathway Genes

The promoters of several flavonoid pathway genes, including *MrLAR, MrANR, MrANS*, and *MrUFGT* were cloned into reporter constructs. Among them, the *MrLAR* and *MrANR* promoters were selected to represent the PA-specific pathway, *MrANS* and *MrUFGT* for anthocyanin-specific pathway. Effector constructs expressing MrMYB6 were assayed with combinations of AtTT8, AtTTG1, MrbHLH1, and MrWD40-1. As shown in Figure 3C, MrMYB6 significantly reduced the activities of *MrLAR* and *MrANR* promoters. This suppression appeared stronger when MrMYB6 was co-transformed with AtTT8 and AtTTG or with MrbHLH1 and MrWD40-1. Similarly, the co-transformation of MrMYB6 with AtTT8 and AtTTG or with MrbHLH1 and MrWD40-1 inhibited the promoters of *MrANS* and *MrUFGT*. However, MrMYB6 alone showed no suppression on the promoters of either *MrANS* or *MrUFGT*.

## Discussion

In higher plants, R2R3-MYB subfamily, including activators and repressors, are major TFs coordinating flavonoid biosynthesis. However, no MYB repressors that regulate flavonoid synthesis have been identified in Chinese bayberry. In this research, we isolated a novel bayberry R2R3-MYB TF, named *MrMYB6*. Protein sequence analysis revealed that MrMYB6 contained two conserved motifs, C1 and C2 (Supplementary Figure 1A), which have been demonstrated to function as repression motifs of subgroup 4 MYB TFs (Jin et al., 2000; Shen et al., 2012). In addition, MrMYB6 shared the LxLxL-type EAR motif and TLLLFR repressor motif with FaMYB1-like proteins which inhibit both anthocyanin and PA biosynthesis (Cavallini et al., 2015; Yoshida et al., 2015) (Supplementary Figure 1A). The phylogenetic analysis clearly placed MrMYB6 in FaMYB1-like clade with co-repressors in anthocyanin and PA biosynthesis, including VvMYBC2 (Cavallini et al., 2015), PtrMYB182 (Yoshida et al., 2015), PhMYB27 (Albert et al., 2014), FtMYB8 (Huang et al., 2019), MtMYB2 (Jun et al., 2015), and FaMYB1 (Aharoni et al., 2001; Paolocci et al., 2011) (Supplementary Figure 1B). Thus, MrMYB6 displayed all structural characteristics of known inhibitors of flavonoid biosynthesis, and might exhibit similar regulatory functions. Moreover, the nuclear localization of MrMYB6 protein supported its potential function as a TF (Figure 1C).

It has been reported that many R2R3-MYB repressors were negatively correlated with the levels of anthocyanins and PAs and the transcripts of related structural genes. For instance, *FtMYB18* was functionally characterized as an inhibitor of anthocyanin and PA synthesis in Tartary buckwheat, and its expression was negatively related to anthocyanin and PA contents (Dong et al., 2020). The elevated expression of *VvMYBC2-L1* during berry development was negatively correlated with the anthocyanin and PA synthesis profiles and also with the expression of *VvDFR, VvLDOX, VvLAR1*, and *VvANR* (Huang et al., 2014; Cavallini et al., 2015). For Chinese bayberry, the soluble PA content gradually decreased, while the anthocyanin content greatly increased during fruit development (Shi et al., 2018a,b). Interestingly, in our present study, *MrMYB6* experienced low expression level exactly at the early developmental stage (57 DAFB) and the harvest stage (113 DAFB), when the soluble PA content and anthocyanin levels were contradictingly the highest (Figure 1B), paralleling with the high expression levels of PA specific *MrANR* gene and many anthocyanin structural genes (Shi et al., 2018a,b). These results indicated that *MrMYB6* expression also exhibited an opposite trend with anthocyanins and PAs accumulation during fruit development in bayberry.

Overexpression of *MrMYB6* in tobacco in our present study resulted in a remarkable loss of red color in flowers due to a significant decrease in anthocyanin levels (Figures 2A,C). However, a recent study suggested that overexpression of gerbera *GhMYB1a* in tobacco plants reduced anthocyanin content but increased the expression of *NtCHS, NtF3H, NtDFR, NtANS*, and *NtUFGT* (Zhong et al., 2020). Ectopic expression of tea CsMYB5a in tobacco resulted into downregulated anthocyanin contents but elevated transcripts of *NtCHS, NtCHI, NtF3H, NtF3*′*H*, and *NtANS* in transgenic tobacco flowers (Jiang et al., 2018). Similarly, our results revealed the transcripts of all anthocyanin structural genes were significantly increased in *MrMYB6*-overexpression tobacco flowers except for *NtCHI* (Figure 2F). *CHI* has been identified as a key gene for anthocyanin biosynthesis. Silencing of *CHI* effectively inhibited anthocyanin accumulation in tobacco (Nishihara et al., 2005). Together with our findings, these results indicated that the inhibition of expression of *NtCHI* played the most important role in declining anthocyanin content in tobacco flowers when overexpressing *MrMYB6* in tobacco. In addition, MrMYB6 was observed to down-regulate the expression of *NtLAR* and *NtANR2*, consistent with the observation that the PA content was reduced in transgenic tobacco flowers (Figures 2D–F). The transcription activity assay further showed that MrMYB6 could significantly inhibit the promoters of *MrLAR* and *MrANR* (Figure 3C). Taken together, these results suggested that MrMYB6 might be a negative regulator for anthocyanin and PA biosynthesis by inhibiting the transcription of related structural genes.

It is well known that flavonoid biosynthesis is transcriptionally regulated by MYB-bHLH-WD40 (MBW) complexes (Ramsay and Glover, 2005). The transcriptional activity of some MYBs require physical interaction with their bHLH and WD40 partners. In some plants, MYB activators and repressors can interact with the same bHLH and WD40 cofactors during regulation of flavonoid biosynthesis. As reported in petunia, repression of anthocyanin biosynthesis by MYB27 required the presence of AN1 (a bHLH protein), which was a necessary composition of the MBW activation complex for pigmentation (Albert et al., 2014). In poplar, MYB115 interacted with bHLH131 and TTG1 (WD40) to promote PA biosynthesis, while PtrMYB57 depended on the same bHLH131 and TTG1 cofactors for negatively regulating anthocyanin and PA biosynthesis (Wan et al., 2017; Wang et al., 2017). Consistent with these reports, our study found that MrMYB6 could interact with MrbHLH1 and MrWD40-1 (Figure 3B), which have been identified as members of the MrMYB1-MrbHLH1-MrWD40-1 complex that positively regulated anthocyanin biosynthesis in Chinese bayberry (Liu, X. et al., 2013; Liu, X. F. et al., 2013). In addition, MrMYB6 could also interact with AtTT8 and AtTTG that are essential for PA accumulation in *Arabidopsis* (Baudry et al., 2004). Meanwhile, our dual luciferase assay data confirmed that the interaction of MrMYB6 with these MBW components increased the inhibitory activity of MrMYB6 on the PA-specific genes such as *MrLAR* and *MrANR* and the anthocyanin-specific genes such as *MrANS* and *MrUFGT* (Figure 3C). Notably, the combination of MrMYB6 with MrbHLH1 and MrWD40-1 exhibited the strongest repression effect on the flavonoid biosynthesis genes compared with other factors. Therefore, these results indicated that MrMYB6 negatively regulated anthocyanin and PA synthesis through directly inhibiting the transcription of flavonoid pathway genes by forming a MrMYB6-MrbHLH1-MrWD40-1 complex. It must be pointed out that without any partners, MrMYB6 displayed no suppression on the promoters of either *MrANS* or *MrUFGT* in anthocyanin-specific pathway (Figure 3C). On the contrary, researches have revealed that several R2R3-MYB repressors such as VcMYBC2, PbMYB120, MaMYB4, and FaMYB1 on their own were sufficient to inhibit anthocyanin accumulation in many fruits such as blueberry, pear, banana, and strawberry (Aharoni et al., 2001; Song et al., 2020; Deng et al., 2021; Li et al., 2021). Therefore, we supposed that there might be other MYB TFs independently down-regulating anthocyanin accumulation in bayberries, which deserved further studies.

In conclusion, a R2R3-MYB transcriptional factor, MrMYB6, which functions as one of the negative regulators for anthocyanin and PA biosynthesis, was isolated from Chinese bayberry. The transcripts of *MrMYB6* displayed a negative correlation with the anthocyanin and insoluble PA accumulation during the late ripening stages of bayberries. Ectopic overexpression of *MrMYB6* caused reduced anthocyanin and PA contents in tobacco flowers because of the decline in the expression of *NtCHI, NtLAR*, and *NtANR2* genes. In addition, MrMYB6 could directly inhibit anthocyanin and PA pathway gene expression by forming MrMYB6-MrbHLH1-MrWD40-1 complex.

## Data Availability Statement

The original contributions presented in the study are included in the article/Supplementary Material, further inquiries can be directed to the corresponding author/s.

## Author Contributions

LS, ZY, and SC were involved in experimental design and data analysis. LS wrote the manuscript. XC, KW, and MY performed most of experiments. WC and SC revised the manuscript. All authors contributed to the article and approved the submitted version.

## Conflict of Interest

The authors declare that the research was conducted in the absence of any commercial or financial relationships that could be construed as a potential conflict of interest.

## Supplementary Material

Supplementary Table 1 Primers used in this study.

Supplementary Figure 1 Sequence analysis of *MrMYB6*. **(A)** Multiple sequence alignment of the R2R3-type motifs in MYB TFs from different plant species. **(B)** Phylogenetic analysis of MrMYB6 and other R2R3-MYB proteins. The phylogenetic tree was constructed using the NJ method with MEGA7 software. The numbers near the branches represents bootstrap value from 1,000 replicates. AmMYB308 (*Antirrhinum majus* MYB308, P81393), SmMYB39 (*Salvia miltiorrhiza* MYB39, AGS55356), GhMYB9 (*Gossypium hirsutum* MYB9, AAK19619), CmMYB1 (*Chrysanthemum morifolium* MYB1, AEO27497), EgMYB1 (*Eucalyptus gunnii*, MYB1 CAE09058), HvMYB1 (*Hordeum vulgare* MYB1, P20026), ZmMYB42 (*Zea mays* MYB42, NP_001106009), ZmMYB31 (*Zea mays* MYB31, NP_001105949.2), PhMYB4 (*Petunia hybrida* MYB4, ADX33331), AtMYB4 (*Arabidopsis thaliana* MYB4, AAC83582.1), AtMYB32 (*Arabidopsis thaliana* MYB32, NP_195225), AtMYB7 (*Arabidopsis thaliana* MYB7, NP_179263), NtMYB2 (*Narcissus tazetta* MYB2, ATO58377), FaMYB1 (*Fragaria* × *ananassa* MYB1, AAK84064.1), PhMYB27 (*Petunia hybrida* MYB27, AHX24372.1), VvMYBC2-L1 (*Vitis vinifera* MYBC2-L1, ABW34393), VvMYBC2-L3 (*Vitis vinifera* MYBC2-L3, AIP98385), FtMYB8 (*Fagopyrum tataricum* MYB8, MK128409), PtrMYB182 (*Populus trichocarpa* MYB182, XP_002305872), MtMYB2 (*Medicago truncatula* MYB2, XP_003616388).
