# Supplementary figures and images for: MrMYB6 From Chinese Bayberry (Myrica rubra) Negatively Regulates Anthocyanin and Proanthocyanidin Accumulation
Source: Front Plant Sci. 2021 Jun 18;12:685654. doi: 10.3389/fpls.2021.685654 (PMC8253226; doi:10.3389/fpls.2021.685654)

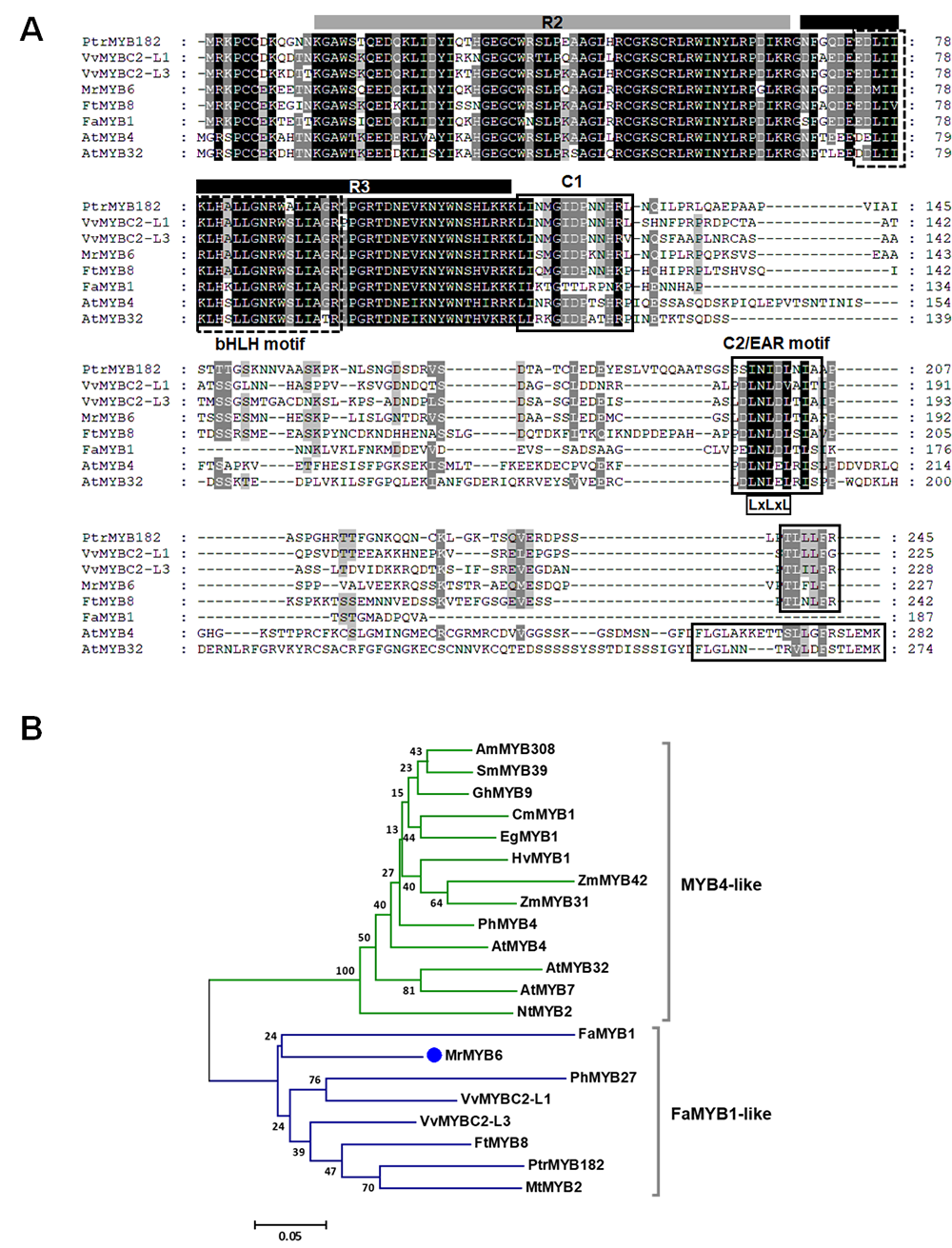

Supplement: Supplementary Figure 1 — Sequence analysis of MrMYB6. (A) Multiple sequence alignment of the R2R3-type motifs in MYB TFs from different plant species. (B) Phylogenetic analysis of MrMYB6 and other R2R3-MYB proteins. The phylogenetic tree was constructed using the NJ method with MEGA7 software. The numbers near the branches represents bootstrap value from 1,000 replicates. AmMYB308 (Antirrhinum majus MYB308, P81393), SmMYB39 (Salvia miltiorrhiza MYB39, AGS55356), GhMYB9 (Gossypium hirsutum MYB9, AAK19619), CmMYB1 (Chrysanthemum morifolium MYB1, AEO27497), EgMYB1 (Eucalyptus gunnii, MYB1 CAE09058), HvMYB1 (Hordeum vulgare MYB1, P20026), ZmMYB42 (Zea mays MYB42, NP_001106009), ZmMYB31 (Zea mays MYB31, NP_001105949.2), PhMYB4 (Petunia hybrida MYB4, ADX33331), AtMYB4 (Arabidopsis thaliana MYB4, AAC83582.1), AtMYB32 (Arabidopsis thaliana MYB32, NP_195225), AtMYB7 (Arabidopsis thaliana MYB7, NP_179263), NtMYB2 (Narcissus tazetta MYB2, ATO58377), FaMYB1 (Fragaria × ananassa MYB1, AAK84064.1), PhMYB27 (Petunia hybrida MYB27, AHX24372.1), VvMYBC2-L1 (Vitis vinifera MYBC2-L1, ABW34393), VvMYBC2-L3 (Vitis vinifera MYBC2-L3, AIP98385), FtMYB8 (Fagopyrum tataricum MYB8, MK128409), PtrMYB182 (Populus trichocarpa MYB182, XP_002305872), MtMYB2 (Medicago truncatula MYB2, XP_003616388). [file Image_1.TIF]
